# Antioxidant activity and protective effect of phyto‐active compounds of *Crataegus azarolus* berries decoction extract against acetic acid‐induced hepatorenal injuries in male rats

**DOI:** 10.14814/phy2.70240

**Published:** 2025-02-09

**Authors:** Houcem Sammari, Anouar Abidi, Saber Jedidi, Nourhen Dhawefi, Hichem Sebai

**Affiliations:** ^1^ Laboratory of Functional Physiology and Valorization of Bio‐Resources, Department of Animal Physiology University of Jendouba, Higher Institute of Biotechnology of Beja Beja Tunisia

**Keywords:** acetic acid, *Crataegus azarolus*, kidney, liver, oxidative stress, rats

## Abstract

The present study evaluated the hepato‐nephronal protective properties of *Crataegus azarolus* berries decoction extract (CAB‐DE) on acetic acid (AA)‐induced oxidative stress and metabolic disorders in rats. Animals (60 rats) were randomly divided into six groups (*n* = 10), with groups 1 and 2 being controls and groups 3, 4, and 5 given increasing doses of CAB‐DE, group 6 were given gallic acid until ulcerative colitis was induced and then intoxicated by an acute intra‐rectal infusion of AA. Our results showed that CAB‐DE‐oral administration had no signs of toxicity or abnormal behavior in rats, with a LD_50_ higher than 3500 mg/kg *bw.* In addition, CAB‐DE protected against AA‐induced nephropathy and hepatic damage in rats, as determined by an increase in organ weights and an alteration in the renal and liver parameters and functions. Moreover, extract co‐administration reduced AA‐induced liver and kidney lipoperoxidation, maintained non‐enzymatic contents such as sulfhydryl groups (‐SH) and reduced glutathione (GSH), and restored antioxidant enzyme activities, including superoxide dismutase (SOD), catalase (CAT), and glutathione peroxidase (GPx). Finally, CAB‐DE might have a possible protective effect against AA‐oxidative stress and dysfunction in the rat liver and kidney, suggesting that *Crataegus azarolus* berries may be beneficial for people suffering from liver issues and nephropathy.

## Graphical abstract

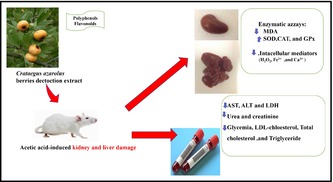

## 1 INTRODUCTION

Ulcerative colitis (UC) is a type of inflammatory bowel disease (IBD) in which relapsing inflammation of the colon and rectum leads to mucosal and submucosal ulcerations and tissue damages that affects quality of life (Danese & Fiocchi, 2011; Dulai et al., 2016).

The latter plays an important role in the development of both systemic inflammatory response syndrome (SIRS) and multiple organ dysfunction syndrome (MODS), considered a factor in multiple organ dysfunction (Carrico et al., 1986; Marshall, 1998).

Importantly, the development of progressive physiological failure in organ systems distant from the starting point of the initial disease process is the most common cause of morbidity and mortality in critically ill patients (Baue, 1975).

Precisely, severe chronic UC induced several extraintestinal manifestations and pathologies, such as acute renal and/or liver failure and liver dysfunction (Caprilli et al., 2000).

To treat the UC, several drugs were used, including anti‐inflammatory such as aminosalicylates, glucocorticosteroids, antibiotics, anti‐TNF‐4, and immunological modulators with documented adverse impacts and cardiovascular, pulmonary, renal, and hepatic complications (Bindu et al., 2020; Curkovic et al., 2013; Rogler, 2010).

Importantly, anti‐inflammatory medications are highly absorbed in the digestive tract. It is metabolized in the liver and transformed into inactive forms in the liver through oxidation. In contrast to other drugs, inactive versions are excreted through the kidneys (Dudhgaonkar et al., 2006). As a result, innovative therapeutic treatments are desperately needed, particularly to reduce the IBDs and other associated complications related to liver and kidney damage.

In the same contrast, several herbal extracts, such as *Terminalia chebula* fruits (myrobalan), were recognized for their capacity to prevent acetaminophen‐induced hepatotoxicity and liver necrosis (Sharma & Rathore, 2010). Among these plants, *Crataegus azarolus* L. (Rosaceae family) is a medicinal plant considered a source that contains an inexhaustible quantity of active substances (Edwards et al., 2012). The plant was employed in traditional medicine (More & White, 2005). *Crataegus azarolus* extracts were used to treat diarrhea (Sammari et al., 2021). They are also known for their regulatory effects against respiratory effects (Beloued, 2005), to treat hydrpopsy (Özcan et al., 2005), and urinary system diseases (Rose & Treadway, 1999).

According to Chang et al. (2005), fruits are used to treat cardiovascular disease, atherosclerosis, and hypertension. In addition, leaf extracts exhibited many beneficial effects, such as antimicrobial, antihyperglycemic, and antihyperlipidemic properties (Abu‐Gharbieh & Shehab, 2017).

As a consequence, the objective of this study was to investigate the hepato‐nephroprotective properties of *Crataegus azarolus* berries decoction extract (CAB‐DE) against acetic acid‐induced oxidative stress and metabolic disturbances in rats.

## 2 MATERIALS AND METHODS

### 2.1 Reagents and chemicals

Ascorbic acid (PubChem CID: 54670067); 2,2‐azino‐bis‐3‐ethylbenzothiazoline‐6‐sulphonicacid (PubChem CID: 5360881); bovine catalase (PubChem CID: 135337101); bovine serum albumin (CAS 9048‐46‐8 | Sigma‐Aldrich); malondialdehyde (MDA) (*PubChem CID:10964*); beta‐carotene (PubChem CID: 10256668); sodium acetate (PubChem CID: 517045); potassium chloride (PubChem CID:4873); aluminum chloride (PubChem CID: 24012); ferrozine (PubChem CID: 34127), butylated hydroxytoluene (PubChem CID: 31404); 5,5‐dithio bis (2‐nitrobenzoic acid) (PubChem CID: 6254); chloroform (PubChem CID: 6212); ethanol (PubChem CID: 57419690); hydrogen peroxide (PubChem CID: 784); epinephrine (PubChem CID: 5816), methanol (PubChem CID: 18177619); potassium dihydrogen phosphate (PubChem CID: 516951); dipotassium hydrogen phosphate (PubChem CID: 24450); 2‐thio‐barbituric acid (PubChem CID: 2723628) and trichloroacetic acid (PubChem CID: 6421) were purchased products from Sigma Chemical Co. (SigmaAldrich GmbH, Steinheim, Germany). All other reagents used were of analytical quality.

### 2.2 *Crataegus azarolus* decoction extract preparation

Mature *Crataegus azarolus* berries were collected in September 2021 from the Ain Draham region, located at the following coordinates: Altitude = 762 m; N 36°44′04.1″, E 008°41′19.8″. The berries are identified by Dr. Imen Bel Haj Ali, Associate Professor at the University of Jendouba. The voucher specimens (No. CA 217) were deposited in the herbarium of the Higher Institute of Biotechnology of Beja. The edible part of the berries was dried at 40°C using an air‐ventilated oven and crushed in an electric blender. The CAB‐DE was prepared by dissolving 1 g of powder in 10 mL of distilled boiled water and maintained under magnetic agitation for 5 min. The product was filtered using Whatman filter paper. The homogenate was lyophilized and freshly used for the phytochemical screening and the in vivo analysis.

### 2.3 Bioactive components determination

The aluminum chloride was used to measure the flavonol levels (Rigane et al., 2011). In short, 1 mL of CAB‐DE, 1 mL of AlCl_3_ (20%), and 3 mL of sodium acetate (50 mg/mL) were combined. Then, the obtained mixture was incubated in the dark for 2 h and 30 min and the absorbance were measured at 440 nm.

The total anthocyanin contents were determined using absorbance differentiation and a buffered solution of (KCl) and (CH_3_COONa). In summary, 0.4 mL of CAB‐DE was mixed with 3.6 mL of KCl and 0.4 mL of CH_3_COONa. The product absorbance was measured after 30 min of incubation at 510 nm (Lee et al., 2005).

The determination of total carotenoids was carried out by the method described by Rønsholdt and McLean (2001). Briefly, 1 mL of the extract was added to 1 mL of hexane, and the absorbance was read at 450 nm. The content of carotenoids was determined in β‐carotene based on the absorbance coefficient of 2500.

### 2.4 Free radical‐scavenging capacity

CAB‐DE's antioxidant activity was evaluated using the ABTS technique (Siddhuraju, 2006). Briefly, 1 mL of diluted extract was mixed with 3 mL of 7 mM ABTS radical solution (ABTS^*•*^+) (PubChem CID: 5360881; 23,681,045) and incubated for 60 min in darkness at room temperature. The absorbance was determined at 734 nm, and the antioxidant capacity of each concentration was measured using the following formula: ((1–A_b_/A_0_) × 100%); with: A_b_ and A_0_ are the absorbance of the samples and the (ABTṠ̇̇̇^*•*^+) solution at 734 nm.

The same concentrations as the test extract were prepared from ascorbic acid and used as a reference molecule. The RSA results that were displayed as IC_50_ values (g/mL) were described as the extract concentration required to scavenge 50% of the (ABTS^*•*^+).

### 2.5 CAB‐DE oral acute toxicity

The oral acute toxicity test of CAB‐DE was established according to the OECD Guideline (2002), to evaluate the safety and efficacy of chemicals. The rats were separated into different groups (*n* = 10). The CAB‐DE was administered orally, and each group was pre‐treated with a single dose. The concentrations were prepared in the following order: 10, 50, 100, 250, 500, 1000, 1500, 2500, and 3000 mg/kg, *b.w*. After treatment, the rats were examined hourly for 24 h to followup on the symptoms of toxicity, respiratory rate, motor coordination, behavior changes, and mortality.

### 2.6 Animals and treatment

Male *Wistar* rats (weighing 210‐230 g) were purchased from the Pharmaceutical Industries of Tunisia (SIPHAT, Tunisia) and housed five per cage. Animals were left to acclimatize for 15 days and were fed with a standard pellet food (Badr‐Utique‐TN) and water ad libitum (12–12 h light–dark cycle; 22 ± 2°C). The rats' experiments were carried out following the local ethics committee of Tunis University for the use and care of animals and in accordance with the NIH recommendations. The protocol has been approved by the “Comite d'Ethique Bio‐medicale (CEBM)” (JORT472001) of the “Pasteur Institute of Tunis.”

**FIGURE 1:**
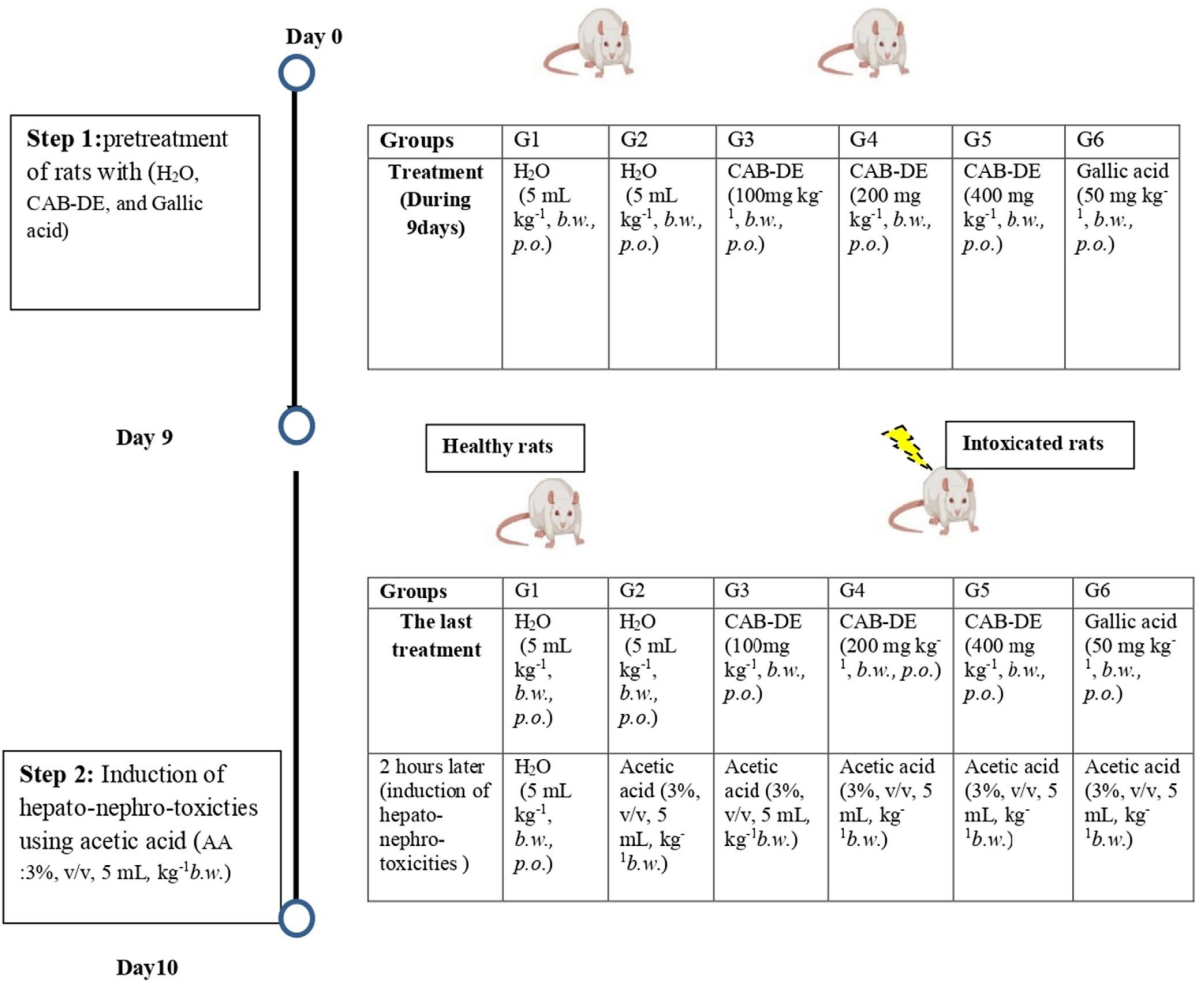
Flowchart of the distribution of animals.

Firstly, rats were divided into 6 groups, and the number of animals used in each group was 10 (*n* = 10) and treated during 10 days following this procedure: groups 1 and 2 served as controls and received distilled water (5 mL kg^−1^, *b.w., p.o*.); groups 3, 4, and 5 were pre‐treated orally with increasing doses of CAB‐DE (100, 200, and 400 mg/kg, *b.w., p.o*.); and group 6 received gallic acid (50 mg/kg, *b.w., p.o*.) until the induction of UC (Figure 1).

Before the last pre‐treatment, all animals were kept fasting for 18 h. Then, 2 hours after the last pre‐treatment, UC was induced in each rat, except group 1. Indeed, a polyethylene tube was inserted through the rectum into the colon for a distance of 8 cm during 30 s, leading to the infusion of acetic acid (3%, v/v, 5 mL, kg^−1^ *b.w*.). 24 h later, rats were anesthetized by intraperitoneal administration of sodium pentobarbital (40 mg/kg, *b.w*.) and sacrificed (Hajji et al., 2018).

Blood was freshly recovered, collected in heparinized tubes, and centrifuged for 15 min at 3000 x*g*. Kidney and liver were quickly excised, homogenized in potassium phosphate buffer solution (30.5 mM K_2_HPO_4_; 19.5 mM KH_2_PO_4_, pH 7.4), and centrifuged for 10 min at 10000 x *g* to recover the supernatant. Finally, those extracts were stored at −20°C and used for biochemical determinations such as MDA, non‐enzymatic antioxidants, H_2_O_2_, free iron, H_2_O_2_ concentrations, and anti‐oxidant enzyme activities. On the other hand, the plasma was processed for the determination of transaminase, alkaline phosphatase (ALP), lactate dehydrogenase (LDH) activities, creatinine, urea, uric acid, C‐reactive protein (CRP), glucose, and intra‐cellular mediators' contents.

### 2.7 Oxidative parameters evaluation

#### 2.7.1 Lipid peroxidation measurement

The hepatic and kidney lipid peroxidation were carried out by the MDA determination, according to the method of Draper and Hadley (1990). The homogenates of the liver and kidney were added to a BHT‐TCA solution (1% BHT (w/v) dissolved in 20% TCA (w/v)) and centrifuged at 1000 x *g* for 5 min. The recovered supernatant was mixed with a buffered solution composed of TBA (120 Mm), Tris (PubChem CID:76001) (26 Mm), and HCl (PubChem CID: 313) (0.5 N). The mixture was heated at 80°C for 10 min. After cooling, the absorbance was determined at 532 nm, and MDA contents were measured using an extinction coefficient for MDA‐TBA of 1.56 × 105 M^−1^ cm^−1^.

#### 2.7.2 H_2_O_2_ determination

The hydrogen peroxide content was determined by the technique of Dingeon et al. (1990). Indeed, H_2_O_2_ reacts with the 4‐aminoantipyrine and the P‐hydroxybenzoic acid to form quinonimine, characterized by a pinkish complex detected at 505 nm.

#### 2.7.3 Enzymatic antioxidant levels

The SOD activities were performed according to Kakkar et al. (1984). Briefly, the kidney and liver homogenates were combined with 10 μL of bovine catalase (0.4 U/mL) and 20 μL of epinephrine (5 mg/mL) and introduced to a buffered sodium carbonate/bicarbonate (PubChem CID: 86624777) (pH 10.2, 62.5 mM) solution. Indeed, the superoxide anion causes the oxidation of epinephrine to adrenochrome. In contrast, SOD decreases the colored complex formation, and the absorbance was recorded at 480 nm.

The CAT activity was determined by detecting the decline of the H_2_O_2_ initial content during 3 min at 240 nm (Aebi, 1984). Summarizing, the enzymatic extracts were added to phosphate buffer (PubChem CID:24978514) (50 mM, pH 7) and 33 mM of H_2_O_2_. The CAT activity was measured using the molar extinction coefficient of 40 mM^−1^ cm^−1^ for H_2_O_2_.

The estimation of glutathione peroxidase (GPx) activities was established according to Flohé and Günzler (1984). The reaction mixture contained 200 μL of liver/kidney enzyme extract, 200 μL of GSH (PubChem CID: 60209673) (0.1 Mm), 200 μL of phosphate buffer (0.1 M, pH 7.4), and 400 μL of H_2_O_2_(5 Mm). The product was incubated for 1 h at 37°C, and 1 mL of TCA (1%) was added to the mixture, which stopped the reaction. The obtained mixture was centrifuged at 3000 × *g* for 10 min. Afterwards, 200 μL of the recovered supernatant was added to 500 μL of phosphate buffer (0.1 M, pH 7.4) and 500 μL of DTNB (10 Mm) solutions. The optical density was evaluated at 412 nm, and the GPx activity was expressed as nmol GSH/min/mg of proteins.

#### 2.7.4 Non‐enzymatic antioxidant contents

The quantification of thiol groups (–SH) was evaluated following the method of Ellman (1959). The renal and hepatic homogenates were mixed with 800 μL of phosphate buffer (0.25 M, pH 8.2) and 100 μL of EDTA (PubChem CID: 13020083) (20 mM). The absorbance was measured at 412 nm (A1).

After that, 100 μL of DTNB (10 mM) was added to the reaction medium and incubated for 15 min at 37°C, and the new value (A2) was determined. The –SH groups content was calculated using deference between A2 and A1 and a molar extinction coefficient of 13.6 × 103 M^−1^ cm^−1^. Concentrations of the –SH groups were determined as nmol of thiol groups per mg of protein.

Renal and hepatic reduced glutathione (GSH) were determined by Sedlak and Lindsay (1968). Indeed, 500 μL of each homogenate were added to (20 mM, pH 4.7) of EDTA, 400 μL of cold distilled water, and mixed with 100 μL of TCA (50%). The mixtures were stirred and centrifuged at 1200 × *g* for 15 min. The 2 mL of recovered supernatant was blended with 400 μL Tris buffer (0.4 Mm, pH 8.9) and 10 μL DTNB (0.01 M). Finally, the absorbance was read at 412 nm.

### 2.8 Biochemical analysis

#### 2.8.1 Assessment of liver function

The liver function parameters such as lactate dehydrogenase (LDH, Ref 13,033), plasmatic aspartate aminotransferase (AST, Ref 10,049) and alanine aminotransferase (ALT, Ref 11,046) were performed using Biomaghreb commercial diagnostic kits (Biomaghreb, Ariana, TN, ISO 9001certificate).

#### 2.8.2 Renal function evaluation

The assessment of serum uric acid (Ref 15,013), creatinine (Ref 25,036), and urea (Ref 20,141) was determined using Biomaghreb commercial diagnostic kits (Biomaghreb, Ariana, TN, ISO 9001certificate).

#### 2.8.3 Inflammatory biomarkers measurement

The alkaline phosphatase (ALP, Ref 13,033) activities and C‐reactive protein (CRP, Ref 45,027) levels were determined using commercially available kits (Biomaghreb, ISO 9001 certification).

#### 2.8.4 Examination of the lipid profile and glucose levels

The following parameters: triglyceride (Ref 20,132), total cholesterol (Ref 20,112), LDL‐cholesterol (Ref 20,113), and glucose (Ref 20,127) were determined using certificated commercial Biomaghreb kits (Biomaghreb, ISO 9001, Tunisia).

### 2.9 Divalent minerals

#### 2.9.1 Free iron measurement

The renal and hepatic free iron were quantified using ferrozine (Leardi et al., 1998). In recap, the iron dissociated from the transferrin‐iron complex by a guanidine acetate solution, then reduced by ascorbic acid, will react with ferrozine, leading to the formation of a pinkish complex determined at 562 nm.

#### 2.9.2 Calcium measurement

The ionized calcium level was evaluated by the procedure of Stern and Lewis (1957). Briefly, the precipitated calcium, as Ca^2+^oxalate, produced a complex with the o‐cresolphthalein, and the absorbance was determined at 570 nm.

### 2.10 Statistical analysis

The data were analyzed by one‐way analysis of variance (ANOVA) using statistical software SAS (Statistics Analysis System, Version 8.2) and expressed as means ± SE. The difference is considered significant when the *p* < 0.05.

## 3 RESULTS

### 3.1 Phenolic compounds and antioxidant capacity

The phytochemical test indicated that CAB‐DE was rich in total phenolic components, as illustrated in Table 1. In fact, CAB‐DE contains substantial levels of flavonols and carotenoids. On the other hand, anthocyanins constituted the least proportion.

**TABLE 1:** Phytochemical screening and ABTS• free radical‐scavenging activity of *Crataegus azarolus* berries decoction extract and ascorbic acid.

Parameters	CAB‐DE
Flavonols (μg RE/g DM)	4.67 ± 1.8
Anthocyanins (mg CG/ g DM)	0.42 ± 0.17
Carotenoids (μg/100 mL)	2.35 ± 0.76
ABTS IC_50_ (μg/mL)	119.83 ± 7.28
Ascorbic acid IC_50_ (μg/mL)	27.03 ± 3.55

*Note*: Data are expressed as mean standard deviation (±SD). Flavonols content expressed as rutin equivalent. Anthocyanins content expressed as cyanidin‐3‐glucoside equivalent. Carotenoids content expressed as μg/mL of extract.

Abbreviations: ABTS, 2,20‐azino‐bis [3‐ethylbenzthiazoline‐6‐sulfonic acid]; DM, dry matter; IC_50_, The sample inhibitory concentration that can reduce the ABTS concentration by 50%.

As shown in Table 1, the antioxidant capacity evaluated by the ABTS technique demonstrates that CAB‐DE extract was characterized by a low IC_50_ (119.83 ± 7.28 μg/mL), despite the fact it is still higher than ascorbic acid.

### 3.2 Acute toxicity test

The oral acute toxicity study in rats indicated that administration of CAB‐DE at increasing doses (10, 50, 100, 250, 500, 1000, 1500, 2500, and 3500 mg/kg, *b.w*.) didn't cause any mortality or affect animal behavior during the entire monitoring period. Consequently, the LD_50_ of CAB‐DE is above 3500 mg/kg *b.w*., *p.o*.

### 3.3 Effect of AA and CAB‐DE on organ weights

The effect of acetic acid, sub‐acute pretreatment with CAB‐DE and gallic acid on liver and kidney weights is illustrated in Table 2. Indeed, AA‐acute intoxication induces a significant increase in kidney and liver mass. According to our results, pretreatment with azerole decoction berries extract and GA considerably (*p* < 0.05) limited the AA‐induced organ weight increase. Interestingly, treatment with CAB‐DE (400 mg/kg, *b.w., p.o*.) showed the most important regulating effect.

**TABLE 2:** Effect of CAB‐DE on AA‐induced increase in liver and kidney weights.

Groups	Liver weight	Kidney weight
Control	6.68 ± 0.56	1.34 ± 0.27
AA	9.02 ± 0.98*	2.06 ± 0.77*
AA+CAB‐DE‐100	8.57 ± 0.70^*#*^	1.84 ± 0.53^*#*^
AA+CAB‐DE‐200	7.89 ± 0.96^*#*^	1.76 ± 0.62^*#*^
AA+CAB‐DE‐400	7.44 ± 0.89^*#*^	1. 45 ± 0.56^*#*^
AA+GA	7.13 ± 0.21^*#*^	1.57 ± 0.39^*#*^

*Note*: Animals were pretreated with CAB‐DE (100, 200, and 400 mg/kg, b.w., p.o.), GA (50 mg/kg, b.w., p.o.), or distilled water for 10 days. After that, they were challenged with a single anal administration of AA (300 mg/kg, b.w.) (3%, v/v, 5 mL kg^−1^ b.w.) or NaCl 0.9% for 24 h. Data are expressed as mean ± SD (*n* = 10); *p < 0.05 compared to the control group, and ^#^ *p* < 0.05 compared to the AA group.

### 3.4 MDA and H_2_O_2_ determination

**FIGURE 2:**
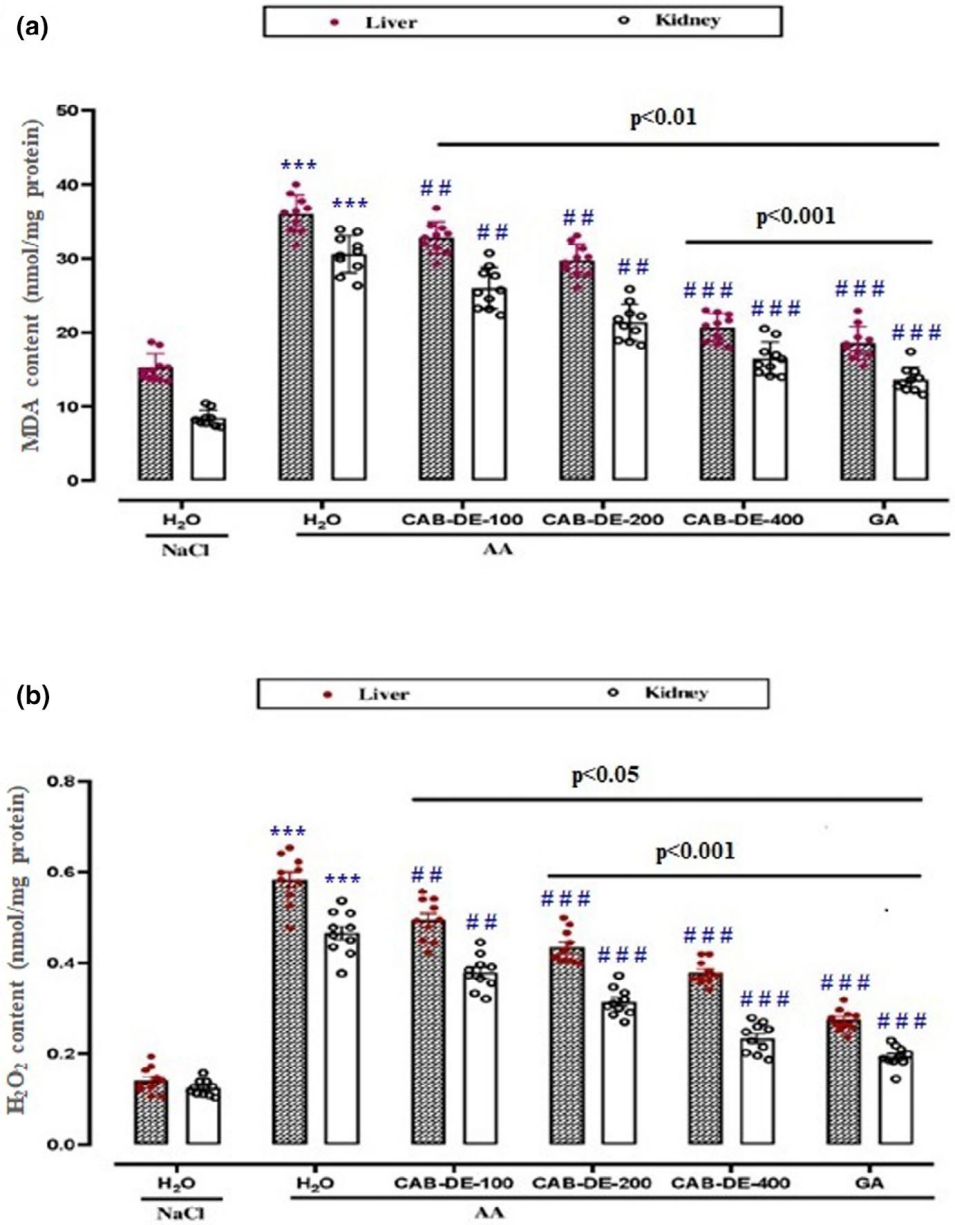
CAB‐DE effect on AA‐induced increase in MDA (A) and H_2_O_2_ (B) levels in liver and kidney rats. CAB‐DE (100, 200, and 400 mg/kg, *b.w*., *p.o*.), gallic acid (50 mg/kg, *b.w., p.o*.), and distilled water were used to pre‐treat the animals during 10 days. For 24 h, rats were challenged with a single anal administration of AA (300 mg/kg, *b.w*.) (3%, v/v, 5 mL kg^−1^ *b.w*.) or NaCl 0.9%. The data are expressed as mean ± SD (*n* = 10). ***p < 0.001 compared to the control group; ^#^ *p* < 0.05, ^##^ *p* < 0.01, and ^*###*^ *p* < 0.001 compared to the acetic acid group. The lines presented a significant difference between the two groups shown.

To study the protective role of CAB‐DE against AA‐induced oxidative stress in the kidney and liver, the homogenates were tested to determine the lipoperoxidation and hydrogen peroxide levels. When compared to the control group, AA induced a significant increase in MDA (Figure 2a) and H_2_O_2_ (Figure 2b) contents. However, CAB‐DE pre‐treatment substantially protected against AA‐induced MDA and H_2_O_2_ augmentation (Figure 2).

### 3.5 Effect of CAB‐DE on AA–induced hepatic and renal antioxidant enzyme activities depletion

**FIGURE 3:**
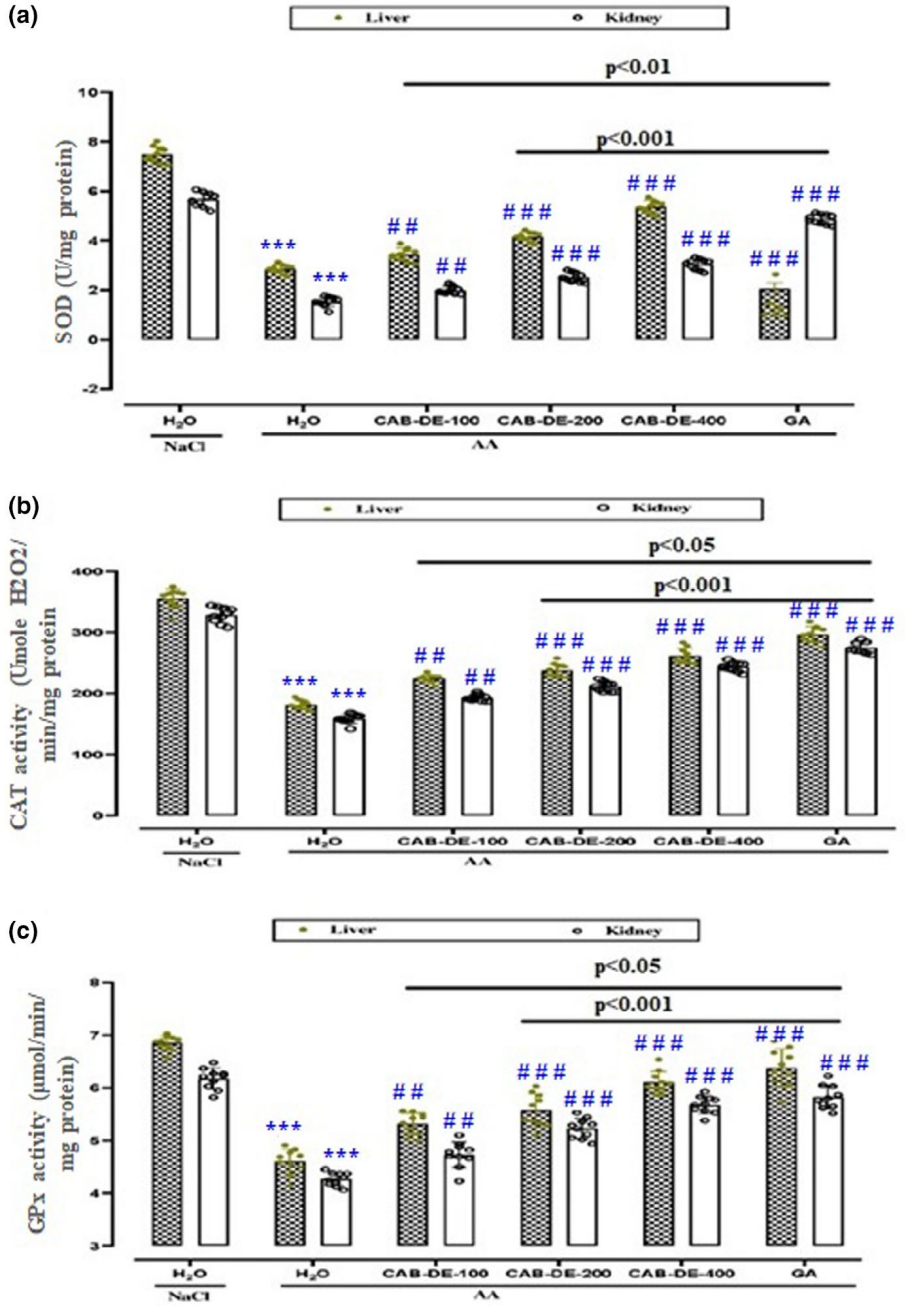
CAB‐DE effect on AA‐induced decrease in CAT (A), SOD (B), and *GPx (C)* activities in liver and kidney rats. CAB‐DE (100, 200, and 400 mg/kg, *b.w*., *p.o*.), gallic acid (50 mg/kg, *b.w., p.o*.), and distilled water were used to pre‐treat the animals during 10 days. For 24 h, rats were challenged with a single anal administration of AA (300 mg/kg, *b.w*.) (3%, v/v, 5 mL kg^−1^ *b.w*.) or NaCl 0.9%. The data are expressed as mean ± SD (*n* = 10). ***p < 0.001 compared to the control group; ^#^ *p* < 0.05, ^##^ *p* < 0.01, and ^*###*^ *p* < 0.001 compared to the acetic acid group. The lines presented a significant difference between the two groups shown.

In the present study, the effects of CAB‐DE and GA pretreatments on kidney and liver antioxidant enzyme activities were examined, and the results are shown in Figure 3. The single intoxication by AA induces a significant (*p* < 0.05) depletion in hepato‐renal antioxidant enzyme activities such as SOD (Figure 3a), CAT (Figure 3b), and GPx (Figure 3c) compared to the control group.

CAB‐DE treatments substantially (*p* < 0.05) restored antioxidant enzyme activities in liver and kidney in a dose‐related manner.

### 3.6 Effect of CAB‐DE and acetic acid on hepatic and renal non‐enzymatic antioxidants

**FIGURE 4:**
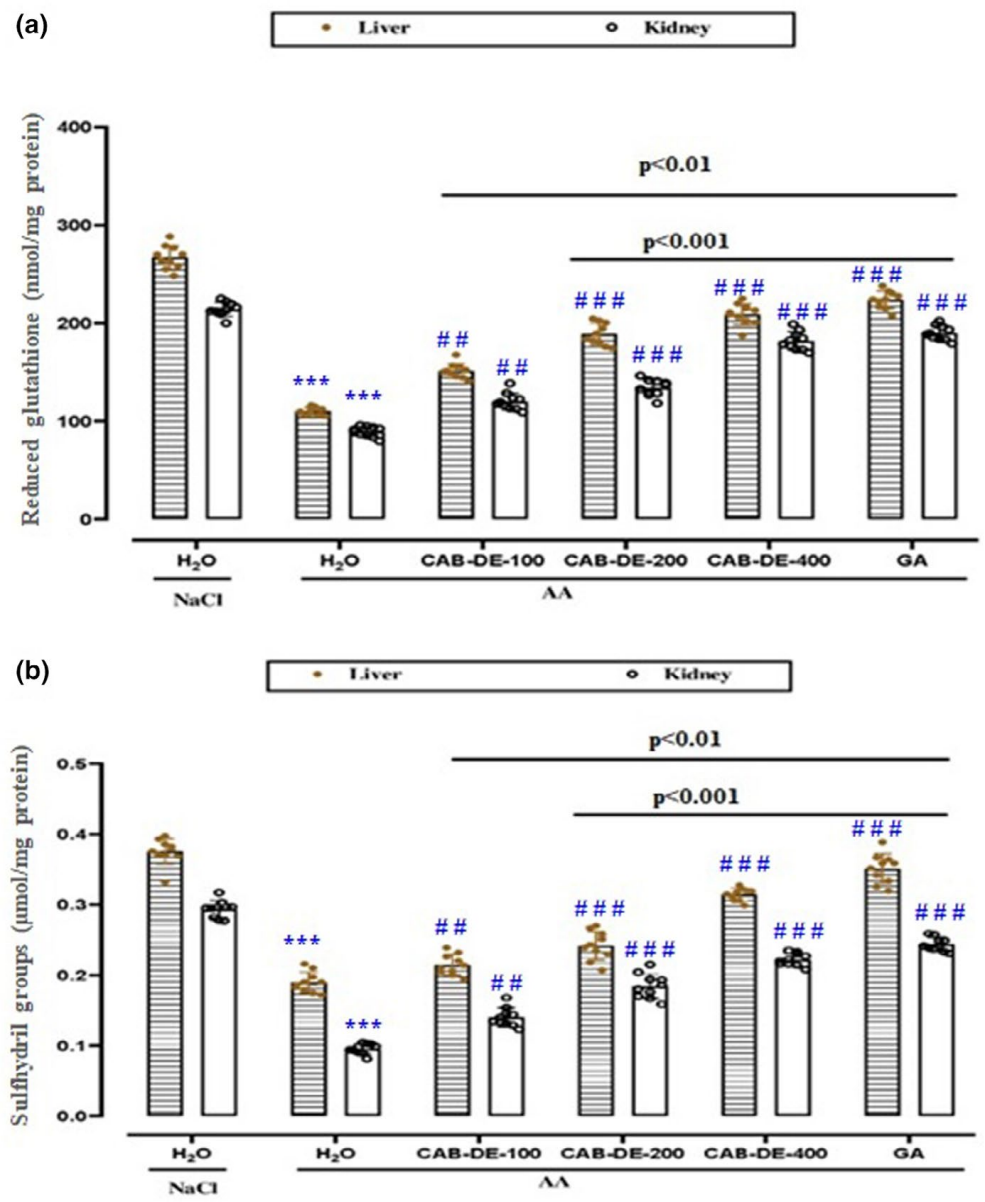
CAB‐DE effect on AA‐induced decrease in reduced glutathione (A) and sulfhydryl groups (B) in liver and kidney rats. CAB‐DE (100, 200, and 400 mg/kg, *b.w*., *p.o*.), gallic acid (50 mg/kg, *b.w., p.o*.), and distilled water were used to pre‐treat the animals during 10 days. For 24 h, rats were challenged with a single anal administration of AA (300 mg/kg, *b.w*.) (3%, v/v, 5 mL kg^−1^ *b.w*.) or NaCl 0.9%. The data are expressed as mean ± SD (*n* = 10). ***p < 0.001 compared to the control group; ^##^ *p* < 0.01, and ^*###*^ *p* < 0.001 compared to the acetic acid group. The lines presented a significant difference between the two groups shown.

Non‐enzymatic antioxidants determination in hepato‐renal tissues showed that AA induced a remarkable depletion of thiol groups (Figure 4a) and reduced glutathione (Figure 4b). As expected, the in vivo antioxidant capacity of the CAB‐DE pretreatment drastically (*p* < 0.05) preserved the content of non‐enzymatic antioxidants in a dose‐dependent manner.

### 3.7 Effect of CAB‐DE on liver function parameters

As shown in Table 3, the single administration of acetic acid (3 mL kg^−1^, *b*.*w*., *p*.*o*.) induced a remarkable deregulation of liver functions expressed by a significant increase in AST, ALT, and LDH plasma activities. Interestingly, sub‐acute pretreatment with CAB‐DE considerably (*p* < 0.05) protected against those metabolic disorders in a dose‐dependent manner.

**TABLE 3:** Effect of *Crataegus azarolus* berries decoction extract pre‐treatment on acetic acid‐induced alterations in liver functions (AST, ALT, and LDH) in rats.

Groups	AST(UI/L)	ALT(UI/L)	LDH(UI/L)
Control	93.65 ± 5.49	46.90 ± 4.18	221.32 ± 18.37
AA	174.23 ± 9.83*	97.42 ± 6.04*	384.68 ± 15.37*
AA+CAB‐DE‐100	162.51 ± 6.28^#^	83.59 ± 7.31^#^	352.47 ± 29.22^#^
AA+CAB‐DE‐200	146.20 ± 7.10^#^	75.94 ± 7.86^#^	316.94 ± 26.36^#^
AA+CAB‐DE‐400	139.87 ± 9.56^#^	68.39 ± 7.53^#^	271.81 ± 25.94^#^
AA+GA	115.43 ± 8.62^#^	59.27 ± 5.70^#^	258.27 ± 23.60^#^

*Note*: Animals were pretreated with CAB‐DE (100, 200, and 400 mg/kg, b.w., p.o.), GA (50 mg/kg, b.w., p.o.), or distilled water for 10 days. After that, they were challenged with a single anal administration of AA (300 mg/kg, b.w.) (3%, v/v, 5 mL kg^−1^ b.w.) or NaCl 0.9% for 24 h. Data are expressed as mean ± SD (*n* = 10); *p < 0.05 compared to the control group, and ^#^ *p* < 0.05 compared to the AA group.

### 3.8 Effect of CAB‐DE on kidney function parameters

More evidently, renal tests demonstrated that AA‐intoxication induced a disturbance of uric acid metabolism, including an increase in plasma urea and creatinine levels. However, pretreatment with CAB‐DE (100, 200, and 400 mg/kg) significantly (*p* < 0.05) re‐established AA‐induced kidney function parameter deregulations (Table 4).

**TABLE 4:** Effect of *Crataegus azarolus* berries decoction extract pretreatment on acetic acid‐induced alterations in kidney functions in rats.

Groups	Urea (mmol/L)	Uric acid (mmol/L)	Creatinine (μmol/L)
Control	6.88 ± 2.10	0.410 ± 0.045	117.32 ± 10.37
AA	9.34 ± 1.23*	0.138 ± 0.012	164.28 ± 12.66*
AA+CAB‐DE‐100	8.95 ± 1.41^#^	0.164 ± 0.053	155.62 ± 16.08^#^
AA+CAB‐DE‐200	8.41 ± 1.65^#^	0.239 ± 0.089	149.11 ± 15.73^#^
AA+CAB‐DE‐400	7.79 ± 0.80^#^	0.302 ± 0.076	142.49 ± 13.51^#^
AA+GA	7.32 ± 1.52^#^	0.341 ± 0.084	137.56 ± 14.29^#^

*Note*: Animals were pretreated with CAB‐DE (100, 200, and 400 mg/kg, b.w., p.o.), GA (50 mg/kg, b.w., p.o.), or distilled water for 10 days. After that, they were challenged with a single anal administration of AA (300 mg/kg, b.w.) (3%, v/v, 5 mL kg^−1^ b.w.) or NaCl 0.9% for 24 h. Data are expressed as mean ± SD (*n* = 10); *p < 0.05 compared to the control group, and ^#^ *p* < 0.05 compared to the AA group.

### 3.9 Effect of CAB‐DE on inflammatory markers

This study's findings showed that AA administration induced an increase in plasma C‐reactive protein and alkaline phosphatase activity as compared to the control group. On the other hand, CAB‐DE co‐administration considerably (*p <* 0.05) restored inflammatory indicator levels in a dose‐dependent manner. Additionally, the treatment with GA showed the most anti‐inflammatory effect (Table 5).

**TABLE 5:** Effect of AA and CAB‐DE on plasma CRP and ALP activity levels in rats.

Parameters	CRP (μg dL^−1^)	ALP(UL^−1^)
Control	0.34 ± 0.05	58.16 ± 8.05
AA	0.99 ± 0.12*	127.23 ± 11.79*
AA+ CAB‐DE‐100	0.83 ± 0.03#	93.58 ± 8.26#
AA + CAB‐DE‐200	0.74 ± 0.14#	80.49 ± 10.55#
AA + CAB‐DE‐400	0.68 ± 0.05#	71.34 ± 9.37#
AA + GA	0.46 ± 0.07#	64.29 ± 6.11#

*Note*: Animals were pretreated with CAB‐DE (100, 200, and 400 mg/kg, b.w., p.o.), GA (50 mg/kg, b.w., p.o.), or distilled water for 10 days. After that, they were challenged with a single anal administration of AA (300 mg/kg, b.w.) (3%, v/v, 5 mL kg^−1^ b.w.) or NaCl 0.9% for 24 h. Data are expressed as mean ± SD (*n* = 10); *p < 0.05 compared to the control group, and ^#^ *p* < 0.05 compared to the AA group.

### 3.10 Effect of CAB‐DE on plasma lipid and glucose concentrations

The effect of AA intoxication on lipidemia and glycemia balance has been investigated. The results showed that AA‐induced colitis in rats was associated with considerable hyperglycemia, as well as an increase in total cholesterol, LDL‐cholesterol, and triglyceride contents. As predicted, the pretreatment with CAB‐DE significantly (*p* < 0.05) reestablished these imbalances in a dose‐dependent manner. Furthermore, a substantial degree of protection (*p <* 0.05) has been recorded in the GA group (Table 6).

**TABLE 6:** Effect of CAB‐DE on AA‐induced hyperglycemia and lipid plasma changes in rats.

Parameters	Control	AA	AA + CAB‐DE‐100	AA + CAB‐DE‐200	AA + CAB‐DE‐400	AA+ GA
Cholesterol (mg/dl)	62.28 ± 7.34	107.32 ± 9.87*	87.14 ± 3.67^#^	80.26 ± 8.34^#^	75.46 ± 8.52 ^#^	96.12 ± 8.73^#^
LDL‐Cholesterol (mg/dl)	33.42 ± 5.22	62.79 ± 5.93*	52.40 ± 6.93^#^	47.56 ± 4.46^#^	41.60 ± 7.35 ^#^	50.30 ± 3.27^#^
HDL‐Cholesterol (mg/dl)	15.05 ± 2.48	21.52 ± 2.77*	14.91 ± 3.15^#^	14.46 ± 2.08^#^	17.31 ± 3.31^#^	24.75 ± 3.84^#^
Triglycerides (mg/dl)	69.08 ± 5.49	115.41 ± 9.14 *	99.19 ± 8.45^#^	91.22 ± 8.60^#^	83.25 ± 4.97 ^#^	105.39 ± 7.23^#^
Glycemia (mmol/L)	6.27 ± 0.53	14.46 ± 1.09 *	10.43 ± 0.62^#^	8.73 ± 0.89^#^	5.30 ± 0.39^#^	12.57 ± 0.66^#^

*Note*: Animals were pretreated with CAB‐DE (100, 200, and 400 mg/kg, b.w., p.o.), GA (50 mg/kg, b.w., p.o.), or distilled water for 10 days. After that, they were challenged with a single anal administration of AA (300 mg/kg, b.w.) (3%, v/v, 5 mL kg^−1^ b.w.) or NaCl 0.9% for 24 h. Data are expressed as mean ± SD (*n* = 10); *p < 0.05 compared to the control group, and ^#^ *p* < 0.05 compared to the AA group.

### 3.11 Effect of AA and CA‐BDE on free iron and calcium contents

We also examined the effect of AA and CAB‐DE on intracellular mediators' levels, such as free iron and ionizable calcium (Table 7). Anal administration of acetic acid caused a considerable decrease in intracellular mediators' contents in hepatic and renal tissues.

**TABLE 7:** Effect of *Crataegus azarolus* berries decoction extract on acetic acid‐induced deregulations in liver and kidney free iron and calcium contents in rats.

Groups	Free iron		Calcium	
	Liver (μmol/mg protein)	Kidney (nmol/mg protein	Liver (μmol/mg protein)	Kidney (nmol/mg protein
Control	94.63 ± 10.58	15.60 ± 2.38	88.53 ± 9.11	1.74 ± 0.12
AA	179.47 ± 15.79*	34.97 ± 5.19*	193 ± 11.76*	2.98 ± 0.23*
AA+CAB‐DE‐100	164.22 ± 13.18^#^	30.44 ± 3.82^#^	178.10 ± 19.24^#^	2.62 ± 0.32^#^
AA+CAB‐DE‐200	150.34 ± 16.27^#^	25.02 ± 3.16^#^	159.11 ± 16.39^#^	2.49 ± 0.28^#^
AA+CAB‐DE‐400	134.62 ± 15.33^#^	21.65 ± 3.75^#^	142.58 ± 13.76^#^	2.23 ± 0.22^#^
AA+GA	118.70 ± 12.29^#^	18.40 ± 2.68^#^	116.41 ± 15.30^#^	1.94 ± 0.24^#^

*Note*: Animals were pretreated with CAB‐DE (100, 200, and 400 mg/kg, b.w., p.o.), GA (50 mg/kg, b.w., p.o.), or distilled water for 10 days. After that, they were challenged with a single anal administration of AA (300 mg/kg, b.w.) (3%, v/v, 5 mL kg^−1^ b.w.) or NaCl 0.9% for 24 h. Data are expressed as mean ± SD (*n* = 10); *p < 0.05 compared to the control group, and ^#^ *p* < 0.05 compared to the AA group.

Importantly, CAB‐DE co‐administration significantly (*p <* 0.05) preserved against these overloads in a dose‐dependent manner, with the highest protection established by 400 mg/kg, *b.w., p.o*. Furthermore, the administration of gallic acid (50 mg/kg, *b.w., p.o*.) also exerted an important protective effect against acetic acid‐induced intracellular mediator's disorder.

## 4 DISCUSSION

In the present study, we investigated the protective properties and the supposed mechanisms of CAB‐DE phenolic compounds against AA‐induced injuries, inflammation, and oxidative stress on the kidney and liver. Firstly, the ABTS test revealed that CAB‐DE has an effective antioxidant capability, indicated by a low IC_50_ value.

The LC‐ESI‐MS analysis of CAB‐DE identified eight phenolic compounds, which are protocatechuic and chlorogenic acid, rutin, quercetin, kaempherol, cirsilineol, and acacetin, of which chlorogenic acid was the main component (Sammari et al., 2024).

Indeed, *Crataegus azarolus* berries extracts were rich in phenolic compounds such as phenolic acids, tannins, flavonoids, anthocyanins, and several antioxidant molecules like carotenoids, xanthophylls, and ascorbic acid, well known for their potent radical scavenging activities (Dursun et al., 2021; Hashempour et al., 2010; Sammari et al., 2021; Sammari et al., 2024).

The in vivo studies showed that the CAB‐DE‐LD_50_ was greater than 3200 mg/kg *bw, p.o*. Indeed, administration of CAB‐DE in increasing and high concentrations did not cause mortality or show any effect on animal behavior. On a similar note, we have demonstrated that the LD_50_ of *Crataegus azarolus* berries aqueous extract was greater than 3500 mg/kg *bw, p.o*. (Sammari et al., 2021).

AA‐intoxication (3%, v/v, 5 mL kg^−1^, *b.w*.) induced an increase in kidney and liver relative weights; however, the tested extract significantly reduced this morphometric deregulation. Our findings are in line with several reports, explaining that several models of chemically induced colitis are often accompanied by an amplification of renal and hepatic mass (Abd El Ghany et al., 2021; Jedidi et al., 2022).

According to Teo et al. (2002), this deregulation in internal organ weight represents an excellent indicator of a substance's toxicity and can be explained by several factors, such as congestion, tissue hypertrophy, or the presence of an inflammatory infiltrate (Betti et al., 2012; Rasekh et al., 2008).

AA administration significantly increased the MDA and H_2_O_2_ contents in hepato‐nephronal homogenates. However, GA or CAB‐DE pre‐treatments substantially and in a dose‐dependent manner reduced the lipid peroxidation levels.

Lipid peroxidation causes severe damage to cells, affecting the fluidity and functions of the membrane (Bryszewska et al., 1995), its increase is directly correlated to pathological conditions (Rizvi & Maurya, 2007). Comparing the level of oxidative stress in both organs, it is noted that the highest level of oxidative stress was reported in the liver. This can be explained by the hepatocyte's membrane composition, which was characterized by a high concentration of polyunsaturated fatty acids and iron (Hamlaoui‐Gasmi et al., 2012).

In the same context, Xu et al. (2023) indicated that the damaging effect of H_2_O_2_ also depends on its implication in the Fenton reaction to produce hydroxyl radicals (HO^•^), known for their highest reactivity.

Another significant feature has been investigated in this paper, describing the oxidative status in hepato‐nephronal tissues related to AA administration. Importantly, CAB‐DE co‐administration restored the antioxidant activities and contents in a dose‐dependent manner. In the same context, it has been shown that experimental ethanol‐induced ulceration was accompanied by a depletion of enzymatic and non‐enzymatic antioxidant levels in the liver and kidney (Jedidi et al., 2022). Overall, phenolic compounds represent an essential xenobiotic with pharmacological importance for human well‐being. Dietary polyphenols have been confirmed to re‐establish redox equilibrium through improving antioxidant enzyme activities such as SOD, CAT, and *GPx* (Bhattacharyya et al., 2014).

Flavanol carotenoids and anthocyanins are recognized for their hepato‐nephroprotective activities; these compounds decrease oxidative stress biomarkers like MDA and raise antioxidant enzymes like SOD and GSH, CAT, and GPx glutathione‐S‐transferase (Akkara et Sabina, 2019; El‐Baz et al., 2021; Orororo et al., 2018; El‐Sayed et al., 2017).

On the other hand, AA administration generated a deregulation in liver parameter functions; this intoxication has been evaluated by an increase in ALT, AST, and LDH activities. Indeed, elevated levels of these enzymes represent a reliable marker of liver status damage and dysfunction (Contreras‐Zentella & Hernández‐Muñoz, 2016), indicating membrane degradation and cytotoxicity (Abdel‐Rahman & Abd El‐Megeid, 2006).

However, CAB‐DE Co‐administration significantly protected against these parameters' deregulation wish is in line with Jaishree et al. (2010), demonstrating that phenolic compound extracts provide protection through liver regeneration and the healing of hepatic cells parenchyma. More specifically, several reports identified the presence of quercetin and rutin (Belkhir, Rebai, Dhaouadi, Congiu, et al., 2013; Belkhir, Rebai, Dhaouadi, Sioud, et al., 2013), in azerole berries, which were recognized for their hepatoprotective properties (Hafez et al., 2015; Thennarasu, 2013).

In the same context, it has been demonstrated that different carotenoid compounds including astaxanthin and *β*‐carotene exerted their ameliorative ability against alanine aminotransferase (ALT), aspartate aminotransferase (AST), total bilirubin, and albumin as liver function biomarkers (El‐Baz et al., 2021).

As expected, our findings showed that AA‐intra‐rectal intoxication induced an imbalance in kidney functions expressed by a depletion of urea and an increase in uric acid and creatinine contents in plasma. The elevated uric acid levels were explained in part by the status of oxidative stress. Indeed, its synthesis increases with the content of oxygen species as a reaction to reduce peroxynitrite (Hooper et al., 1998). The study of Ranganathan et al. (2013) in mouse model colitis revealed that inflammation was found to have a key role in triggering kidney damage. This was confirmed by several histopathological investigations (Mohamed et al., 2019), showing structural changes in renal glomerular capillaries and tubular injuries AA‐induced.

It has been shown that anthocyanins normalized kidney function and decreased the surface of glomerular lesions and fibrosis scores in the same environment. The modulation of amino acid metabolism in the kidney may be one of the fundamental processes of anthocyanins (Li et al., 2020).

The intriguing topic concerning CAB‐DE's anti‐inflammatory potential has also been examined in this investigation. In fact, CAB‐DE dramatically lowered ALP activity and CRP production. According to a recent study by Kallassy et al. (2017), the phenolic contents of *Crataegus azarolus* have been linked to a number of mechanisms of action for their anti‐inflammatory properties. These mechanisms include the inhibition of pro‐inflammatory cytokines expression, such as IL‐1*α*, IL‐1β, and IL‐6, and the transcription of pro‐inflammatory chemokines, such as CCL3 and CCL4.

The obtained results showed that the co‐administration of CAB‐DE to rats intoxicated with AA resulted in a significant increase in serum levels of triglycerides, LDL‐cholesterol, and total cholesterol. The extract's potential for hypolipidemia, which has been previously documented by other investigations (Al Humayed, 2017; Mostafa et al., 2018), may account for the current findings.

The study of Belkhir, Rebai, Dhaouadi, Congiu, et al. (2013) manages to identify several phenolic components in *Crataegus azarolus* berries, which are vitexin, B2 procyanidin, and chlorogenic acid. These compounds well known for their hypolipidemic properties and regularizing effect on lipidemia metabolism, involving various pathways. These consist of total cholesterol and LDL‐cholesterol contents reduction, increasing the number of hepatic LDL receptors, and inhibiting intestinal acyl‐coenzyme A transferase activity (Chu et al., 2003; Lin et al., 2011; Meng et al., 2013). The putative involvement of glycemia in acetic acid‐induced colitis and CAB‐DE activity were studied. Data indicated that AA‐induced colitis implicated an hyperglycemia in rats. However, CAB‐DE pretreatment exerts a regulating effect against this metabolic disorder which are in line with the study of Sammari et al. (2021). In the same context, *Crataegus azarolus* leaves extract demonstrated an hypoglycemic effects in alloxan diabetes mince, wish are polyphenols and flavonoids responsible for α‐glucosidase inhibition (Abu‐Gharbieh & Shehab, 2017). These substances have the ability to raise hepatic glycogen production and storage while decreasing insulin resistance (Xin et al., 2021).

More interestingly, the single administration of AA (5 mL kg^−1^ *b.w*., *p.o*.) induced renal and hepatic intoxication is complemented by a significant increase in free iron contents in the hepatocytes and kidney tissues. The dysregulation of (Fe^2+^) homeostasis can induce several cell damages by stimulating the Fenton reaction (Xu et al., 2024), and also accelerate the lipid peroxidation (Elmastaş et al., 2006).

CAB‐DE administration significantly reduced the release of free iron in liver and kidney homogenates in a dose‐dependent manner. Indeed, herbal antioxidants have the ability to chelate and inhibit the release of transition metals (Elekofehinti & Kade, 2012). Thus, previous studies have indicated that quercetin and rutin effectively block the free iron Fe^2+^ release in cerebral homogenates (Omololu et al., 2011).

This experiment showed that the AA colitis model was accompanied by an increase in Ca^2+^ concentrations in renal and hepatic tissues. This homoeostasis disruption was explained by the oxidizing circumstances and massive production of reactive oxygen species (ROS) (Jedidi et al., 2022). The outflow of Ca^2+^ may lead to mitochondrial electron chain transport deregulation and cell necrosis (Paillard et al., 2013).

## 5 CONCLUSION

In conclusion, the present research suggests that pretreatment with CAB‐DE may be beneficial in the prevention of hepato‐nephronal dysfunction as an extra intestinal disorder triggered by the experimental AA‐colitis model. These capacities were explained partly by CAB‐DE properties, which were proven in minimizing oxidative stress, inflammation, and metabolic biomarker deregulations. Finally, azerole berries can be used as an herbal supplement preserving against hepatic and kidney injuries.

## AUTHOR CONTRIBUTIONS

**Houcem Sammari** and **Abidi Anouar**: Carried out the experiments, analyzed the data, and wrote the article. **Saber Jedidi** and **Nourhen Dhawefi:** Participated in the processing and analysis of the data. **Hichem Sebai**: Contributed in final revision of the manuscript. All authors read and approved the final manuscript.

## FUNDING INFORMATION

The author(s) received no financial support for the research, authorship, and/or publication of this article.

## CONFLICT OF INTEREST STATEMENT

The authors declare that they had no conflicts of interest with respect to their authorship or the publication of this article.

## ETHICS STATEMENT

The NIH guidance and the local Tunis University ethics committee for the use and care of animals were followed in all maintenance and sacrifice procedures. The protocol was approved by the “Comité d'Ethique Bio‐medicale (CEBM)” (JORT472001) of the “Institut Pasteur de Tunis.”

## Data Availability

The data that support the findings of this study are available on request from the corresponding author. The data are not publicly available due to privacy or ethical restrictions.
